# Genetic and Landscape Connectivity of Blacklegged Ticks During Range Expansion in Select States of the Midwestern USA

**DOI:** 10.1002/ece3.72360

**Published:** 2025-10-22

**Authors:** Dahn‐young Dong, Susan M. Paskewitz, Jean I. Tsao, Sean D. Schoville

**Affiliations:** ^1^ Department of Integrative Biology University of Wisconsin‐Madison Madison Wisconsin USA; ^2^ Department of Entomology University of Wisconsin‐Madison Madison Wisconsin USA; ^3^ Department of Fisheries and Wildlife Michigan State University East Lansing Michigan USA; ^4^ Department of Large Animal Clinical Sciences Michigan State University East Lansing Michigan USA

**Keywords:** genetic connectivity, *Ixodes scapularis*, landscape ecology, Midwest, population expansion, population genetics

## Abstract

Since the 1970s, the Midwestern USA has experienced an expansion of blacklegged ticks ( *Ixodes scapularis* ), the primary vector of Lyme disease caused by *Borrelia burgdorferi* , leading to increased Lyme disease incidence. Public health surveillance indicates that Northwestern Wisconsin has served as refugia for these ticks, seeding an expansion into neighboring states such as Michigan. However, the process of re‐emergence and invasion remains unclear. To improve tick management, we examine whether environmental variables in the Midwestern (eastern North Central) region have constrained tick dispersal and whether connectivity corridors can be identified. By developing fine‐scale spatial population genomic data, our analyses reveal genetically diverse populations in Wisconsin, with northern populations contributing to recent expansions within the state. We identify several east–west gene flow corridors facilitating tick dispersal in Wisconsin. An independent dispersal network exists along Wisconsin's Mississippi River, extending southwards. In contrast, Michigan populations exhibit sharp genetic divergence from Wisconsin and Indiana populations, with low genetic diversity and high in‐state gene flow. We also identify high landscape connectivity in the region connecting the Michigan Peninsulas and significant gene flow at the landmass near southern Lake Michigan. Geographical isolation, as well as landscapes with low soil humidity during summer and high human disturbance, were found to limit gene flow across the region, although these effects were minor. Management of blacklegged ticks in the region can be enhanced by recognizing that landscape connectivity has influenced the dispersal of distinct genetic populations, and targeted interventions in seemingly less tick‐favorable landscapes may help mitigate the spread.

## Graphical abstract

Blacklegged ticks ( *Ixodes scapularis* ), vectors of Lyme disease, have expanded across the Midwestern USA since the 1970s, with at least three genetically structured populations. Fine‐scale genomic and landscape analyses reveal distinct gene flow corridors and barriers, highlighting how environmental connectivity shapes tick range expansion and informing targeted management strategies.

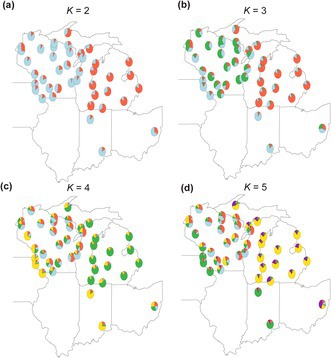

## 1 Introduction

Since the 1970s, the Midwestern USA has been impacted by the re‐emergence and range expansion of the northern, host‐seeking blacklegged tick ( *Ixodes scapularis* ) ecotype that has increased regional Lyme disease incidence (Eisen and Eisen 2023; Gardner et al. 2020; Lantos et al. 2017). The blacklegged tick, especially the northern ecotype, is the primary vector of the Lyme disease‐causing bacterium, *Borrelia burgdorferi sensu stricto*, and several other critical human diseases. An increase in disease incidence usually follows the arrival and establishment of the ticks (Eisen and Eisen 2018).

**FIGURE 1:**
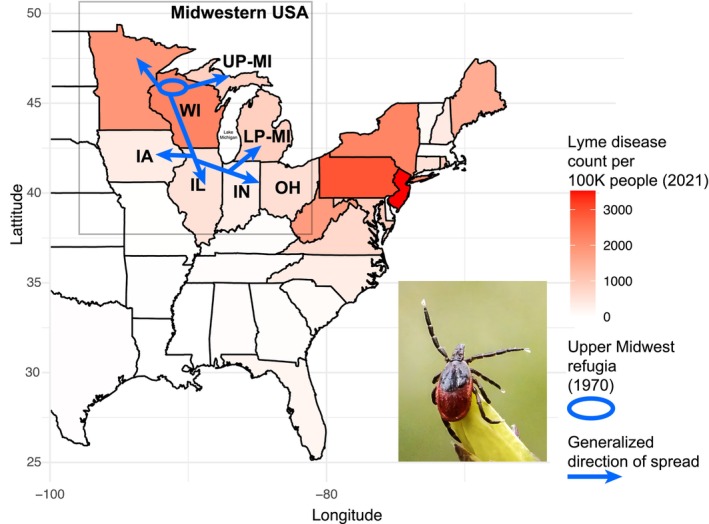
A map of select states in the Eastern United States with Lyme disease incidence in 2021, per 100,000 people, color‐coded by state. *Ixodes scapularis* is widespread in the mapped region, but the Northern ecotype, in particular, is responsible for most Lyme disease incidence. The gray box bounds the Midwestern United States. Surveillance records indicate that the Midwestern USA has experienced a re‐emergence of the independent *Ixodes scapularis* Northern ecotype from Upper Midwest refugia since the 1970s. Since then, the Northern ecotype has been spreading within the Midwestern USA following the generalized direction of spread, with Michigan still undergoing active invasion at the time of the study. The states marked with acronyms constitute the focal region of research. Wisconsin (WI), Upper Peninsula Michigan (UP‐MI), Lower Peninsula Michigan (LP‐MI), Wisconsin—Iowa border (WI–IA), Illinois (IL), Indiana (IN), and Ohio (OH). The incidence count source is from the Centers for Disease Control and Prevention's WONDER database (Centers for Disease Control Prevention 2021). The refugia and direction of spread are adapted from Eisen and Eisen (2023). The female adult *Ixodes scapularis* photograph, embedded on the lower right of the figure, is courtesy of Graham Hickling, used with permission.

Surveillance data show that the re‐emergence of blacklegged ticks from Northwestern Wisconsin started during the 1970s and 1980s (Figure 1) due to reforestation efforts and possibly a warming climate (Eisen and Eisen 2023). Gradually, the invasion progressed throughout the western half of Wisconsin and then continued eastward, leading to a statewide invasion during the 1990s and early 2000s. Since the 1990s, the neighboring state of Michigan has undergone similar invasion patterns, with ticks first expanding in areas closer to the west, namely, Wisconsin and Indiana, and gradually invading central and eastern counties of Michigan (Centers for Disease Control and Prevention 2025; Lantos et al. 2017; Walker et al. 1998). Although surveillance data document county‐by‐county range expansion, which facilitates vector control, they may not accurately describe connectivity patterns and dispersal mechanisms.

A key challenge is to describe and identify ancestral structure, gene flow, and landscape determinants that mediate blacklegged tick dispersal over the Midwestern landscape, which will provide insight into range expansion dynamics and increasing tick‐borne disease risk (Mechai et al. 2016; Swei et al. 2020). Population and landscape genomics provide a useful framework for understanding the relationship between genetic variation and landscapes (Hemming‐Schroeder et al. 2018; Manel et al. 2003). Driven by dispersing populations over generations, genomes will record patterns of neutral variation, primarily the product of gene flow and genetic drift, considering that mutations are rare and selection is specific to small regions of the genome (Schoville et al. 2012). The extent of genetic differentiation across populations sampled across regions, if spatially correlated, can reveal the trend of range expansion and the extent of dispersal (Bohonak 1999). Moreover, neutral genetic differentiation can be studied across populations to identify geographic and landscape factors that have facilitated or impeded gene flow over time, such as landscape cover type and configuration, climatic conditions, and habitat quality (Balkenhol et al. 2017). If functional barriers impede the spread of vectors, management programs can be implemented to potentially limit disease transmission in some regions (Hemming‐Schroeder et al. 2018). These functional barriers may exist or be enhanced in uninvaded regions to prevent disease emergence.

The earliest research on genetic differentiation among blacklegged ticks focused on continental‐scale diversity patterns and found a deep genetic divergence between northern and southern ticks (Humphrey et al. 2010; Norris et al. 1996; Sakamoto et al. 2014). With a worsening Lyme disease epidemic, more recent work has focused on gene flow and the spread of ticks in the Northeastern USA Genetic variation and diversity patterns were used to elucidate dispersal routes in the Northeast, and evidence was found for directional gene flow resulting from both proximal and long‐range dispersal (Khatchikian et al. 2015; O'Keeffe et al. 2022). It has become clear that the genetic structure of tick populations in the Northeast follows a gradient due to an isolation by distance (IBD) pattern resulting from restricted gene flow of ticks among nearby states (Xu et al. 2020). A spatial invasion of the Northeast is favored over an alternative model of local re‐emergence from existing cryptic refugia (Khatchikian et al. 2015). While the existing literature has previously identified environmental influences on the habitat suitability and abundance of blacklegged ticks, it has not been clear that these factors influence genetic connectivity across the landscape (O'Keeffe et al. 2022; Talbot et al. 2020). Because the northern ecotype dominates both the Northeastern and the Midwestern regions, recent comparative genomic studies began to investigate the population genetics of the Midwestern blacklegged ticks in more detail (Frederick et al. 2023; Schoville et al. 2024). While Midwestern ticks are genetically similar to Northeastern ticks (Frederick et al. 2023; Van Zee et al. 2015), recent range expansion events in the Midwest are considered independent from the Northeast (Humphrey et al. 2010). Despite numerous research efforts, the limited spatial sampling density in these studies does not allow for testing detailed range expansion hypotheses and landscape connectivity in the Midwestern region.

Surveillance data suggest that local refugia in northwestern Wisconsin might have seeded the Midwestern expansion (Eisen and Eisen 2023); however, this hypothesis remains untested genetically. The dispersal dynamics from refugia in the Midwest should be evident in genetic data, depending on the number of dispersing blacklegged ticks via host networks from refugia (Røed et al. 2016). Ecological variation is also likely to constrain tick establishment, persistence, and dispersal in this region, such as the availability of favorable forest, soil conditions, and climatic envelope (Diuk‐Wasser et al. 2006; Guerra et al. 2002; Killilea et al. 2008; Larson et al. 2022). The current distribution niche modeling suggests that much of the upper Midwest landscape is suitable for blacklegged tick establishment and range expansion (Burtis et al. 2022; Hahn et al. 2016). Nonetheless, it is reasonable to expect that these landscapes will create some spatially heterogeneous gene flow barriers, although limited, for blacklegged ticks in the region. To further our understanding of the interactions between dispersal, establishment, genetics, and the environment, we conducted a more comprehensive spatial sampling of blacklegged ticks in the Midwest. Using population genomic samples for a large set of individuals, we set out to test the hypotheses that (1) the expansion of blacklegged ticks in Wisconsin is from northwestern local refugia within Wisconsin as surveillance data suggested, while (2) the recent expansion in Michigan is derived originally from tick populations in Wisconsin as ecological research suggested. Furthermore, due to the rapid expansion of ticks in the Midwestern region, we test the hypothesis that (3) connectivity is driven primarily by geographical distance and not principally explained by landscape variation.

## 2 Materials and Methods

### 2.1 Sample Collection

**FIGURE 2:**
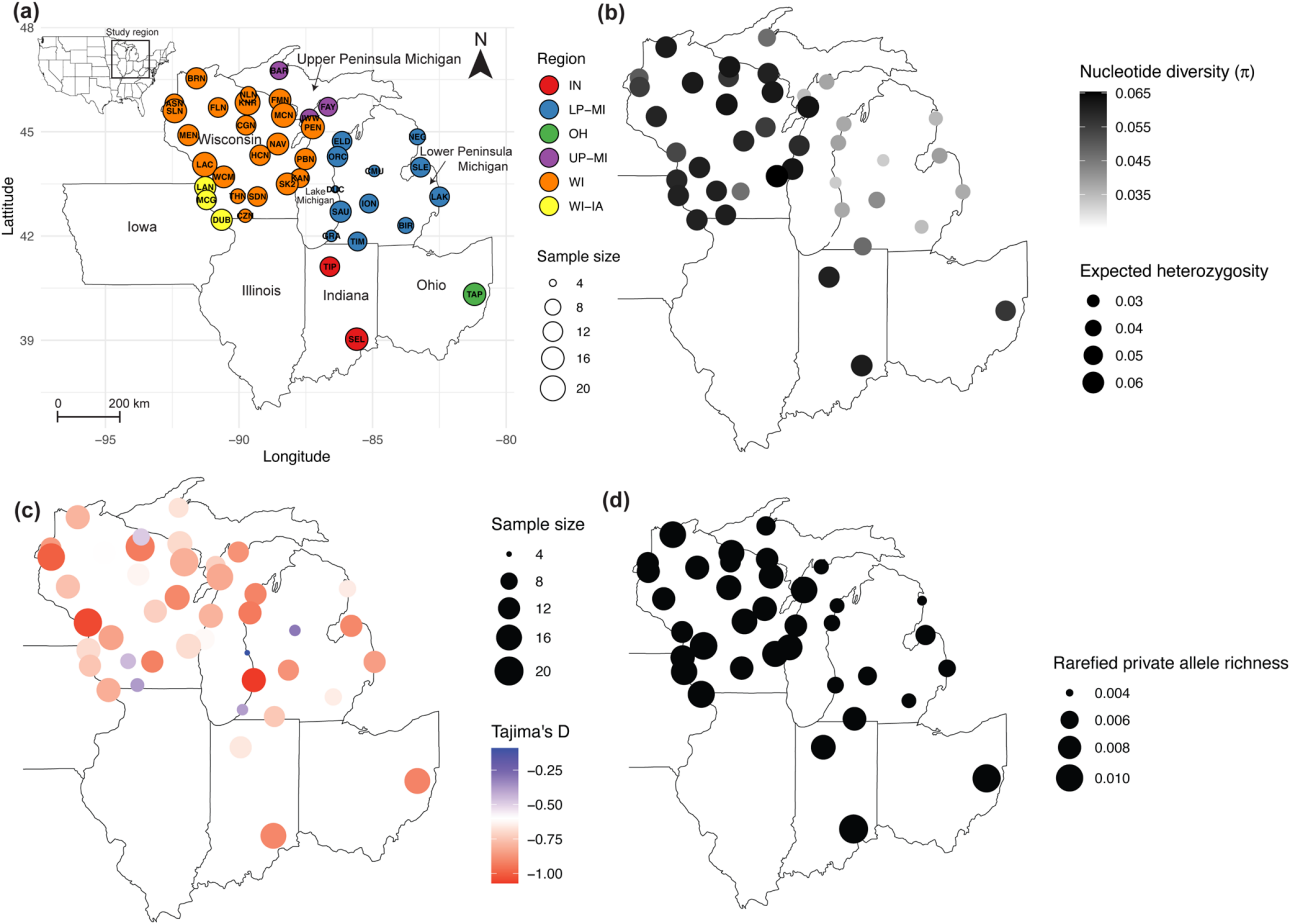
Genetic summary statistics of sampled populations over the study area. (a) A map with sampling sites, i.e., designated populations, indicated by circles. The populations are colored by sampling region. The size of the circles indicates sample sizes. Full site names are listed in Table S1. Wisconsin (WI), the Upper Peninsula of Michigan (UP‐MI), the Lower Peninsula of Michigan (LP‐MI), the Wisconsin–Iowa border (WI–IA), Indiana (IN), and Ohio (OH) are represented. (b) Nucleotide diversity and expected heterozygosity. The larger and darker (more black) sites are more diverse in both indices. (c) Tajima's D, when negative in value, can indicate population expansion. The bigger and redder the circles are, the more negative Tajima's D and the more pronounced the effect of recent population expansion. (d) Rarefied private allele richness, with larger circles, indicates higher proportions of private alleles found in that population alone. Rarefied private alleles were calculated after removing populations with fewer than eight samples.

Questing (host‐seeking) blacklegged ticks (both nymphs and adults) were collected from 2021 to 2023 throughout the Midwestern region of the USA (referred to in some literature as the North Central region). The sampling effort focused on Wisconsin, which recorded the earliest re‐emergence and range expansion, and Michigan as the most recently invaded region. At the same time, we extended the sampling effort to other populations of Midwestern ticks in the Mississippi River watershed at the Iowa and Wisconsin border and more southern regions in Indiana and Ohio. These populations may represent blacklegged tick refugia in the past unglaciated region (Dalton et al. 2022) or refugia that might have persisted despite deforestation through the 1800s (Eisen and Eisen 2023). Multiple individuals were sampled at each site by dragging (Falco and Fish 1992). We collected samples at 21 sites in Wisconsin, 15 sites in Michigan, and 6 sites in other neighboring states. We included all 42 sampling sites in our study, comprising a total of 517 individuals as the final sample set after quality control. The 42 populations are grouped by geographic regions to facilitate discussion, namely Wisconsin (WI), Upper Peninsula Michigan (UP MI), Lower Peninsula Michigan (LP MI), Wisconsin–Iowa Border (WI–IA), Indiana (IN), and Ohio (OH) (Figure 2a, Table S1).

### 2.2 Genomic Data Processing

Blacklegged ticks were either diced with a razor blade prior to extraction or pulverized with pestles by liquid nitrogen (Hamer et al. 2010). Genomic DNA was then extracted using DNeasy Blood and Tissue kits (Qiagen LLC). All extracted ticks underwent a genotype‐by‐sequencing procedure (GBS) using a double‐digest of restriction enzymes (PstI/MspI) to reduce genome complexity (Elshire et al. 2011) conducted at the University of Wisconsin Biotechnology Center. Blacklegged tick samples were individually barcoded and prepared for sequencing using the Illumina DNA Prep Kit (Illumina Inc.). Illumina pair‐end sequencing was conducted on the Novaseq6000 and X Plus platforms. Results were cross‐checked to ensure consistency. Nymphal ticks from Michigan with low DNA input underwent an additional whole‐genome amplification (REPLI‐g) step. Previous work has shown this method to be unbiased for population genomic analysis (Blair et al. 2015). To ensure this, we tested the genotype concordance of single nucleotide polymorphism (SNP) calls using two control samples (sequenced with and without genome amplification).

Raw sequence data was demultiplexed and assigned to individual IDs according to barcodes using *PROCESS_RADTAGS* in *STACKS* 2.64 (Catchen et al. 2013), where the restriction enzyme check was disabled to preserve the most reads. Adapters were removed using *CUTADAPT* 3.5 (Martin 2011), while *TRIMMOMATIC* 0.39 (Bolger et al. 2014) was used to remove low‐quality bases and short reads (LEADING: 3, TRAILING: 3, SLIDINGWINDOW: 4:20, MINLEN: 50). Read quality checks were conducted using *FASTQC* v0.11.9 (Andrews 2019). Paired‐end reads were then aligned to the blacklegged tick reference genome (NCBI: GCF_016920785.2) using *BWA‐MEM* 0.7.17‐r1188 (Li 2013). The aligned reads were converted to a binary file format and sorted using *SAMTOOLS* 1.16.1 (Li et al. 2009). The sorted aligned reads were then used to call SNPs using *GATK 4.4.0.0* (Van der Auwera and O'Connor 2020), essentially following best practices detailed in the Germline Short Variant Discovery pipeline. Variants' missingness was minimized by removing samples with high missingness (99%), as measured by *PLINK2* v2.00a3 SSE4.2 (Chang et al. 2015). The resulting variants were further filtered using VQSR‐informed hard filters per the recommended protocol (QualByDepth < 2, RMSMappingQuality < 40, FisherStrand > 60, StrandOddsRatio > 3, MappingQualityRankSumTest < −12.5, ReadPosRankSumTest < −8).

Finally, the variant call format (VCF) file was further processed to keep only biallelic SNPs present in at least 80% of the samples, using *VCFTOOLS* 0.1.17 (Danecek et al. 2011). The average variant depth (DP) is 29 (Min DP ≈ 6, Max DP ≈ 129, standard deviation SD ≈ 8). As linkage equilibrium can bias some downstream analyses, we removed correlated SNPs using *PLINK2* (window size 50 kb, step size 10, *R* ^*2*^ threshold 0.1). Most analyses require zero missing data, so the remaining missing variants were imputed in *BEAGLE* v5.4 (Browning et al. 2018). We employed an initial principal component analysis of the samples to identify and remove one sample that was a genetic outlier (> 6× standard deviation from the mean). Supporting scripts have been made available at Dryad (Dong et al. 2024). We retained singleton alleles (≈27% of total variants) for downstream analyses as they are informative about invasive processes and have high quality in our study (mean DP coverage ≈ 29, Min DP ≈ 6, Max DP ≈ 124, and SD ≈ 8). As minor alleles are conventionally filtered in molecular ecology studies, we compared our results with a variant set where singletons were removed (minor allele count; MAC = 2) for demonstration purposes (Figure S1).

### 2.3 Genetic Distance Statistics and Isolation by Distance

To characterize genetic differences among the 42 populations across the sampled Midwestern region, we calculated and compared several pairwise genetic distances in *R* 4.3.1 using *ADEGENET* 2.1.10, *GRAPH4LG* 1.8.0, and *MMOD* 1.3.3 (Bowcock et al. 1994; Jombart 2008; Savary et al. 2021b; Winter 2012). Populations with spatially limited gene flow may exhibit IBD patterns, where pairwise genetic distances positively correlate with geographical distances (Wright 1943). “1 – proportion of shared alleles” (a.k.a. Dps) and linearized Fst were chosen for further analyses because they are standard metrics in landscape genetics and exhibited a high correlation to geographic distance (Table S2) (Savary et al. 2021a). Furthermore, Dps is known to be sensitive to more recent gene flow than Fst, so it will be necessary for our study of recent dispersal and range expansion (Savary et al. 2021a). We tested IBD using Mantel tests in the R package *VEGAN* 2.6–4, using great‐circle distance calculated with the function *st_distance* in the R package *SF* 1.0–16 as a measure of geographical distance (Oksanen et al. 2013; Pebesma 2018; Pebesma and Bivand 2023), and generated *R* ^2^ values using the *LM* function in the R package *STATS* 4.3.1 (R Core Team 2023). A multivariate Mantel correlogram was computed for the genetic distances to obtain the strength of IBD at each distance class and to estimate the distance threshold where genetic similarity wanes and IBD begins.

### 2.4 Genomic Summary Statistics

Genomic diversity indices often provide insight into gene flow and population dynamics. Expected heterozygosity, nucleotide diversity, rarefied private allele richness, and Tajima's D (Tajima 1989) were calculated to summarize patterns of genetic variation across the 42 populations defined previously using *POPULATIONS* in *STACKS 2.64* (Catchen et al. 2013), *ADZE‐1.0* (Szpiech et al. 2008), and *DADI* (Gutenkunst et al. 2009). The first two indices describe raw patterns of genetic diversity, while private allele richness measures the extent of gene flow impacting the signal of unique genetic variants, and genome‐wide Tajima's D indicates recent population expansion (if negative) or contraction (if positive). Private alleles were calculated after removing a subset of the 42 populations with fewer than eight samples.

### 2.5 Population Structure

To identify patterns of population ancestry, we employed principal component analysis (PCA) and used two clustering algorithms, *SNMF* (sparse non‐negative matrix factorization) (Frichot et al. 2014) and *CONSTRUCT* 1.0.5 (continuous structure) (Bradburd et al. 2018). PCA is a non‐parametric dimension reduction tool to visualize genetic differences among individuals. The software *PLINK2* was used to impute missing variants among SNP calls and calculate principal component eigenvectors (Chang et al. 2015). Clustering algorithms can identify the best‐fitting number of putative ancestral populations (*K*) and the proportions of different genomic ancestry for each individual. Using the R package *LEA* 3.12.2 (Gain and François 2021), the statistically best‐fit model among *K* = 1 and *K* = 7 possible ancestral population clusters was evaluated by determining the model with the lowest cross‐entropy, and individual ancestry proportions were then estimated under that model using the *SNMF* function. The estimated ancestries of individual samples were combined at the 42 sampling locations to display the spatial pattern of genomic ancestry. However, ancestry estimation can be confounded by IBD patterns, which can cause an overestimation of the number of ancestral populations (Frantz et al. 2009). Therefore, *CONSTRUCT* was employed as a control method to estimate ancestry proportions while correcting for IBD. This Bayesian method evaluates the predictive accuracy of spatial (IBD‐corrected) and non‐spatial models (*SNMF*‐like) (Bradburd et al. 2018), where cross‐validation of different *K* values (1–7) and (non)spatial models was performed to find the best model. After cross‐validation, the best model was run with 200,000 steps of the Markov chain Monte Carlo (MCMC) procedure to stabilize posterior estimates for ancestry estimation, and this was repeated in two independent runs.

### 2.6 Barriers and Connectivity of Gene Flow

Because complex gene flow patterns could arise due to the re‐emergence of ticks from refugia or rapid range expansion, we implemented fast estimation of effective migration surfaces (*FEEMS*), which can reveal spatial patterns of accumulated gene flow (Marcus et al. 2021). In short, the migration pattern across discrete spatial samples was estimated on graph edges as a migration surface to help infer time‐averaged regional migration barriers and connectivity. *FEEMS* implements a Gaussian Markov Random Field model under a penalized likelihood framework. Different lambda values that serve as a smoothing regularization parameter were evaluated in the leave‐one‐out cross‐validation framework, and the best lambda value was selected as the one that produced the least cross‐validation error.

Genetic graph networks are an alternative approach to characterizing gene flow patterns and can be used to reveal dispersal networks by using the summary statistic Dps, which contains signatures of more recent gene flow. We estimated dispersal networks from the genetic data using *GRAPH4LG*. This software implements a rule‐based minimum spanning tree pruning method and fast greedy modularity optimization to generate the genetic graph with modules (Savary et al. 2021b). Node sizes were measured proportionately to the mean of the inverse weights of the links connected to each node, which is a good proxy for comparing site‐level dispersal (Koen et al. 2016).

### 2.7 Landscape Ecological Data Collection and Processing

**FIGURE A1:**
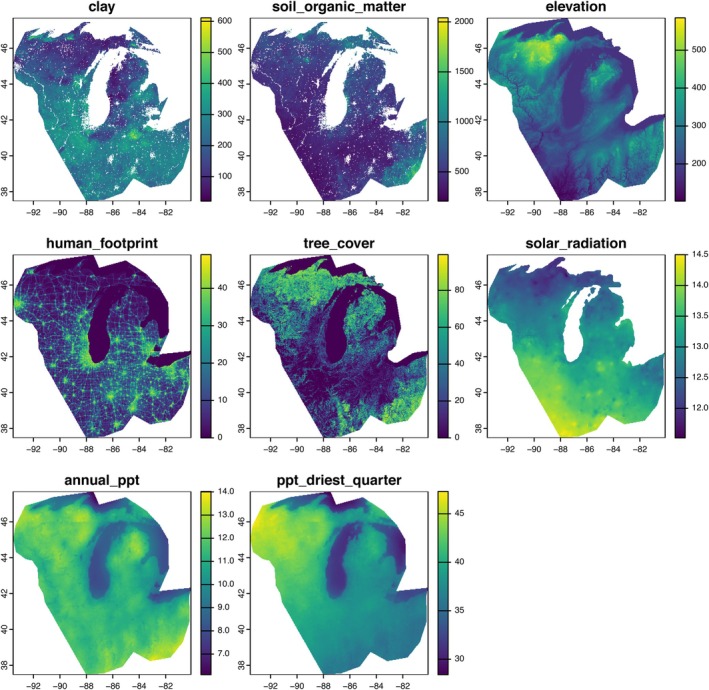
Landscape ecology data showing the range of variation and spatial heterogeneity of the included variables in the Midwestern USA.

To examine the effect of the landscape factors on gene flow, landscape ecological data hypothesized to be relevant for tick ecology (Boeckmann and Joyner 2014; Burtis et al. 2019; Killilea et al. 2008) were collected from public sources, including climate data from WORLDCLIM (Fick and Hijmans 2017), human development index (Venter et al. 2016), soil data from SOILGRIDS (Poggio et al. 2021), 30‐year normal solar radiation (horizontal) from PRISM (Prism Climate Group 2011), and forest cover from the NLCD 2021 USFS Tree Canopy Cover dataset (USDA Forest Service 2023). First, highly correlated variables were removed (threshold > 0.7) while keeping the solar radiation variable related to desiccation risk on microclimate (Eisen et al. 2006). After variable selection, the final variable set (Figure A1) includes elevation, precipitation of the driest quarter, annual precipitation, human development index, 15‐year average soil clay and organic matter content at 0–5 cm depth, 30‐year normal solar radiation (horizontal), and forest cover. All final variables were converted to raster format under the same geographic projection (NAD83), extent (Midwestern USA), and resolution (30 arcseconds or approximately 1 km^2^) with the R package *TERRA* 1.7.78. Sampling locations were converted to spatial objects using *SF* 1.0–16. Spatial locations with missing raster cell values were sampled from their nearest neighbors.

### 2.8 Isolation by Resistance (IBR) and Environment (IBE) Analyses

While straight‐line geographical distance is often used as a predictor for genetic differentiation due to spatially limited gene flow (IBD), the landscape matrix can limit gene flow through a process known as isolation by resistance (IBR) (McRae 2006). To test for IBR, landscape features are assigned costs and interpreted as biologically relevant factors that impede genetic flow between pairs of populations. In addition to IBD and IBR, isolation by environment (IBE) is a third process where habitat characteristics at the sampling sites can lead to genetic differentiation between sites (Wang and Bradburd 2014), such as when similar or dissimilar site habitat conditions influence dispersal rates between sites.

IBR was modeled using all landscape variables (Figure A1) by optimizing resistance values using the observed genetic distance calculated by linearized Fst, a preferred indicator for accumulated landscape impacts on genetic diversity (Schleimer et al. 2023). Landscape data were aggregated to an approximately 10 km resolution for more efficient optimization. IBR modeling was conducted using maximum likelihood estimation in the R package *RADISH* with “maximum likelihood population effects” (MLPE) regression to account for population effects (*n* = 42) (Clarke et al. 2002; Peterman and Pope 2021). Here, the best‐supported model among all landscape variable combinations was evaluated using AIC and against the IBD null model, and the coefficients of each modeled landscape factor were obtained. Model *R* ^2^ values were calculated using the *lm* function. Next, we took the best‐supported IBR conductance model from *RADISH* and converted it to a resistance surface. The resistance surface is then used by *OMNISCAPE* v0.6.2 to visualize landscape‐informed gene flow (block size = 3, radius = 300 km, r_cutoff = 3rd quartile of each resistance surface) (Landau et al. 2021; McRae et al. 2016). The *OMNISCAPE* input file is the best‐fit raster resistance model generated by *RADISH* but rescaled with a minimal value of 1 to conform to the program requirements.

To evaluate IBE, multiple matrix regression with randomization (MMRR), implemented in the R package *ALGATR* 1.0.0 (Chambers et al. 2023), was used while accounting for overall landscape geography (either IBD or IBR) (Wang 2013). Specifically, either the geographical distance matrix or the resistance matrix generated from the best IBR model was used with IBE in the regression framework to co‐estimate effects on genetic distance (the response variable). We used a randomized permutation (*n* = 999) approach to account for population dependency and calculate the statistical significance of the IBE models. All landscape variables were tested in the model without further variable selection, as the selection was done in the upstream data preparation stage. Model *R* ^2^ values were calculated to assess the overall variation explained by the regression.

To control for any potential bias from deeper historical population structure, which might skew the results of landscape models, we additionally used linear mixed‐effects models (LMM) with the R package *corMLPE* 0.0.3 and *NMLE* 3.1–162 to add a random effect that controls for *K* = 3 on the best‐supported IBR model and IBE models (Pinheiro 2011; Pinheiro and Bates 2000). Each pair of populations was assigned to a unique ancestral pair ID (Moreno‐Contreras et al. 2023). To compare across models, we calculated AIC_*C*_, implemented in the R package *MUMIN* 1.48.4 (Bartoń 2024), to select the best model for interpretation.

## 3 Results

### 3.1 Genetic Distance, IBD, and Summary Statistics

The bioinformatic processing resulted in ~80 K unlinked SNPs representing all geographical regions and samples (17% missingness on average and later imputed, mean depth ≈ 29, Min depth ≈ 6, Max depth ≈ 129, standard deviation ≈ 8) (Table S3). We retained all high‐quality singletons for analyses as their removal using the MAC filter did not impact the interpretation of results. Specifically, population genetic summary statistics that are sensitive to MAC only shifted values marginally, and the relative difference across populations and regions remains the same as the analyses that kept all alleles (Figure S1). Genetic distance measures were generally low among populations, suggesting low genetic differentiation, but the magnitude varied depending on the statistics (Table S4). To select the best representative genetic distance metrics, we examined cross‐correlation (Table S5) and correlation with geographical distance (IBD) using Mantel tests (Figure S2, Table S2). Both Dps (mantel coefficient = 0.21) and linearized Fst (0.28) provide moderately strong and significant signals for geographic isolation across the Midwestern USA. The *R* ^2^ value for the IBD model with linearized Fst is 8% (Table 1). Mantel correlograms using the selected genetic distances revealed that the pattern of IBD is weak but leads to genetic differences at scales of about 300 km, indicating that the effect of dispersal attenuates at larger geographical scales (Figure S3).

**TABLE 1:** Model comparison of landscape hypotheses: Isolation by distance, isolation by resistance, and isolation by environment in explaining the genetic variation of blacklegged ticks in the study region, measured by linearized Fst.

Landscape genetic models with genetic distance linearized Fst as the response variable	*R* ^2^ Genetic variance explained by the landscape models	Significant landscape predictors with coefficients interpreted as increase (+) or decrease (−) gene flow	AIC_*C*_ after using linear mixed‐effect model to account for ancestral population structure
Isolation by distance (IBD) alone (null model)	8%	Site differences in: Geographical distances (−)	−6862.484
Isolation by resistance (IBR) alone	27%	Areas with: Soil organic matter (+), clay content (+), human footprint (−), solar radiation (+), precipitation of driest quarter (+)	−6865.065
Isolation by environment (IBE) while co‐estimating IBD	21%	Site differences in: Geographical distances (IBD) (−), soil organic matter (−), elevation (−), annual precipitation (−)	−6731.268
Isolation by environment (IBE) while co‐estimating IBR	33%	Site differences in: Resistance distance (IBR) (−), solar radiation (+), elevation (−)	−6733.292

*Note:* The *R* ^*2*^ values of each model and the gene flow interpretation of each significant landscape variable are presented. Variables with positive signs indicate the promotion of gene flow. Detailed IBD models are reported in Table S2 and Figure S2. Detailed IBR models are reported in Table S6, and detailed IBE models, while accounting for either IBD or IBR, are reported in Tables S7 and S8, respectively. Model selection is based on AIC_*C*_, and the IBR model with the lowest AIC_*C*_ is the best model.

Expected heterozygosity (H_e_), nucleotide diversity (π), and rarefied private allele richness show spatial variation across states, with Wisconsin, Indiana, and Ohio exhibiting greater genomic diversity and rarefied private allele richness than Michigan (Figure 2b,d). Tajima's D (Figure 2c) had a less pronounced spatial trend across states, but all populations have negative values, suggesting that populations are expanding. Note that Tajima's D estimates are less reliable when sample sizes are small, and thus, values at sites with fewer than eight samples should be interpreted with caution.

### 3.2 Population Structure

**FIGURE A2:**
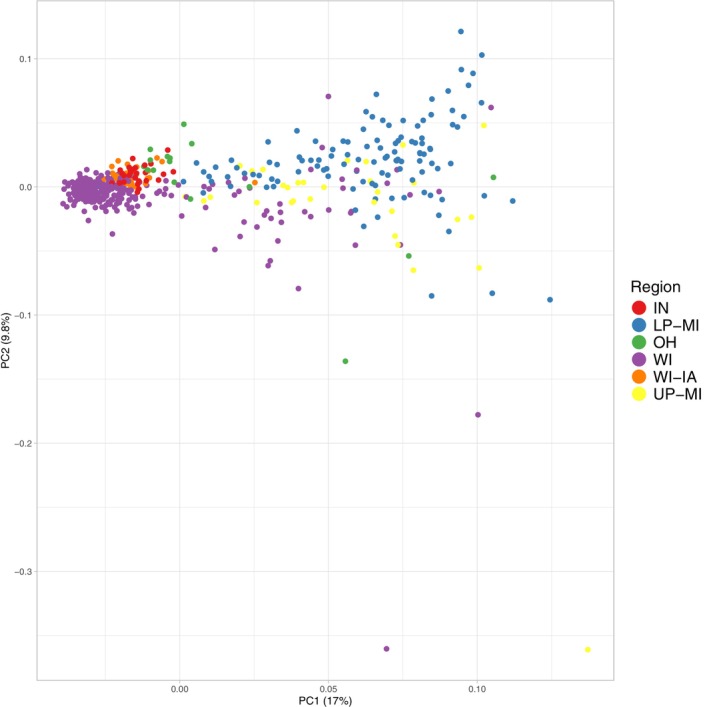
Principal component analysis of individual blacklegged tick genetic samples collected from Wisconsin (WI), Upper and Lower Peninsula Michigan (UP‐MI and LP‐MI), and putative source areas along the Wisconsin–Iowa (WI–IA) border, Indiana (IN), and Ohio (OH) (Figure 2a). Individual samples are color‐coded based on sampling region.

The genomic diversity among individual samples was estimated by PCA and visualized using the first two principal components (Figure A2). The general trend across PCs shows that almost all sites in Wisconsin are clustered into one region of the graph, with some individual samples scattered divergently from the main cluster. Wisconsin sites with divergent samples along PC1 are not geographically clustered. Michigan samples were grouped along PC1, with some overlapping with the divergent individuals from Wisconsin. All individual samples from the Mississippi River watershed along the Wisconsin–Iowa border (WI–IA), Indiana (IN), and Ohio (OH) seem to cluster together. They are distinct from the majority of Wisconsin and Michigan samples. Ohio contains a few samples that overlap with Wisconsin and Michigan in PC space.

**FIGURE 3:**
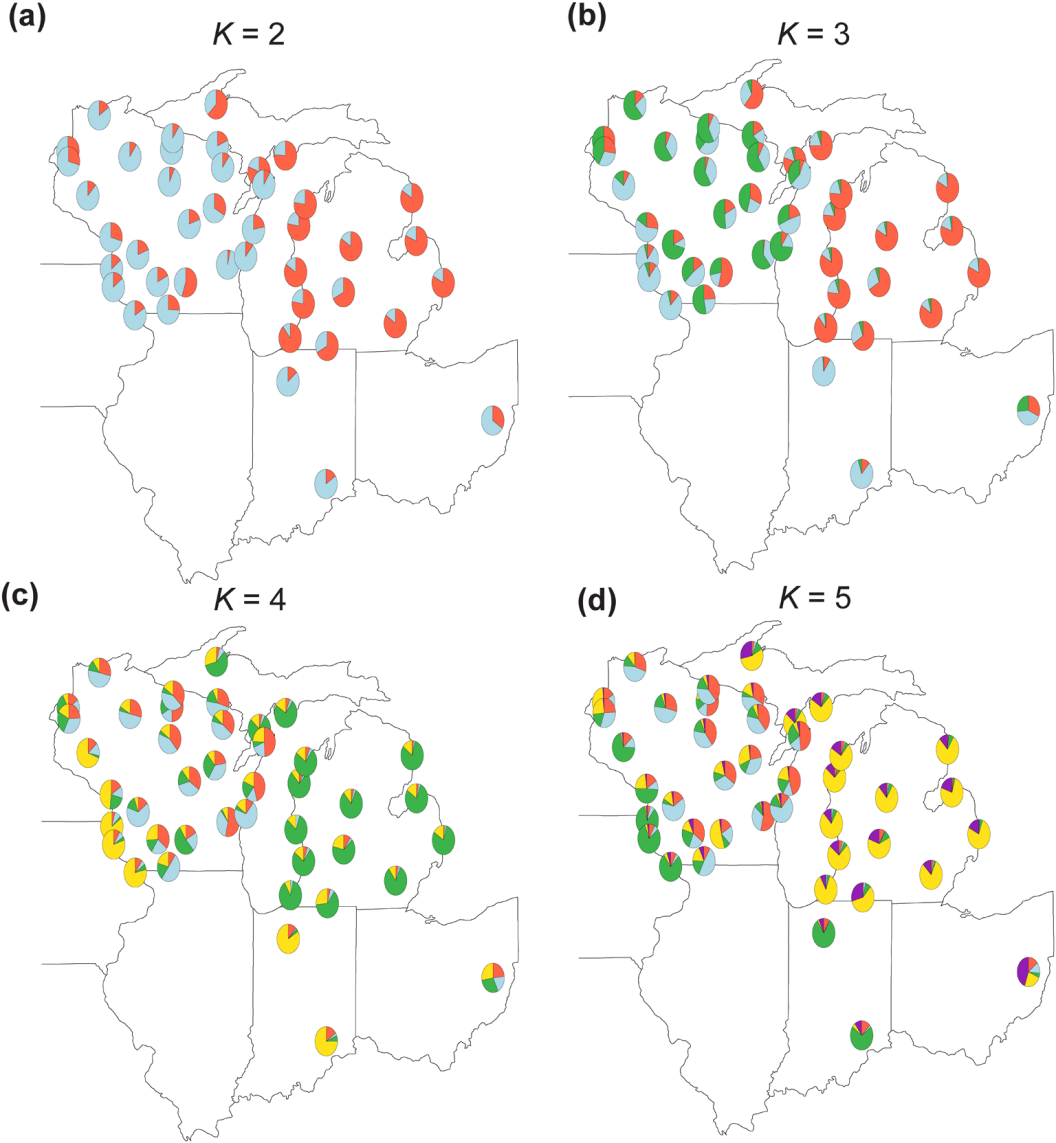
Pie charts visualizing admixture proportions. (a–d) *K* = 2–5 shows unique ancestry in MI relative to other regions, with a notable difference from WI. (b–d) *K* = 3–5 show distinctive patterns of ancestries among the WI–IA and IN populations, represented by the dominant blue color in (b) *K* = 3. The pattern persists for (c–d) *K* = 4 and 5. WI ancestry is further subdivided as *K* increases in value. OH is diverse in its ancestry. (MI: Michigan, WI: Wisconsin, IA: Iowa, IN: Indiana, OH: Ohio).

**FIGURE A3:**
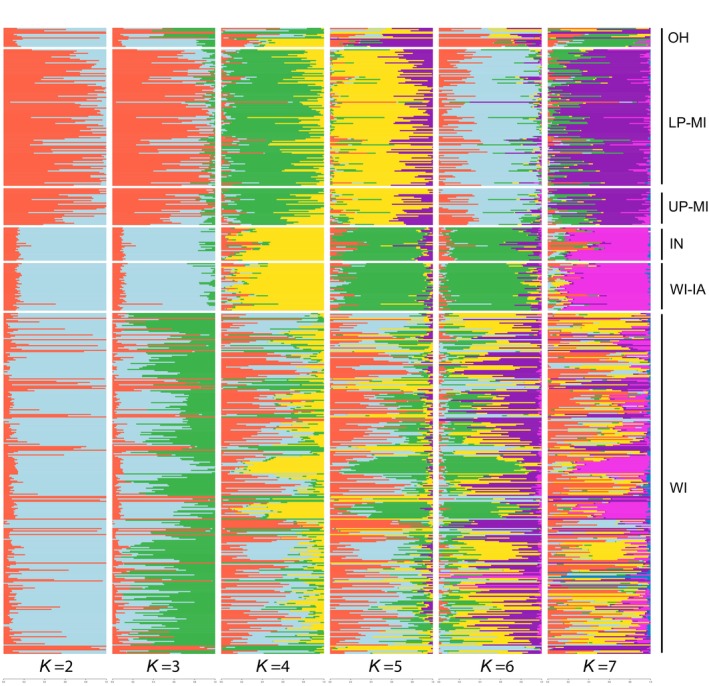
Panels of *SNMF* ancestry proportion plotted for the models of *K* = 2 to K = 7 ancestral population clusters from left to right. Each row is one sample, and the samples are ordered alphabetically by population designations (Table S1). The samples are further grouped by the acronyms of the sampling regions. Each sample contains multiple colors representing the different inferred ancestries with various proportions.

Genetic structure was further quantitatively explored by estimating individual genomic ancestry using the *SNMF* clustering approach. The best model was estimated as *K* = 2 and the second best as *K* = 3 (Figure S4). We examined bar plots of *K* = 2 to *K* = 7 to assess the interpretability of different putative ancestries. Individuals from the same geographical regions show similarity in ancestry proportions but are heavily admixed (Figure A3). The ancestry proportions were further examined by aggregating individuals into sample sites and representing their ancestry as pie charts (Figure 3). While *K* = 3 is the most visually interpretable, Wisconsin is defined by the most diverse ancestries regardless of *K* values. Michigan's homogeneity stands out across *K* values. Meanwhile, the analysis consistently distinguishes the populations from Indiana, Iowa, and southwestern Wisconsin from all other sites in Wisconsin and Michigan. All populations and regions exhibit degrees of admixture (Figure 3).

Lastly, to corroborate the *SNMF* results by accounting for spatial autocorrelation, we conducted *CONSTRUCT*. Results show that the spatial and non‐spatial models perform similarly well in predictive accuracy from *K* = 3 through 7 (Figure S5a). To further select the best model among *K* = 3 and 7, we examined the ancestry layer contribution at various *K* values. The spatially corrected model at *K* = 3 has all ancestries contributing meaningful proportions (1%) (Figure S5b) while keeping *K* parsimonious. This *CONSTRUCT*'s spatial model *K* = 3, which corrects for spatial autocorrelation, produces similar visual results to the admixture pattern found in *K* = 3 of *SNMF* (Figures S5c, 3b). Therefore, the concordance of results between *SNMF* and *CONSTRUCT* confirms the robustness of the *SNMF K* = 3 plot.

### 3.3 Gene Flow and Landscape Isolation Modeling

**FIGURE 4:**
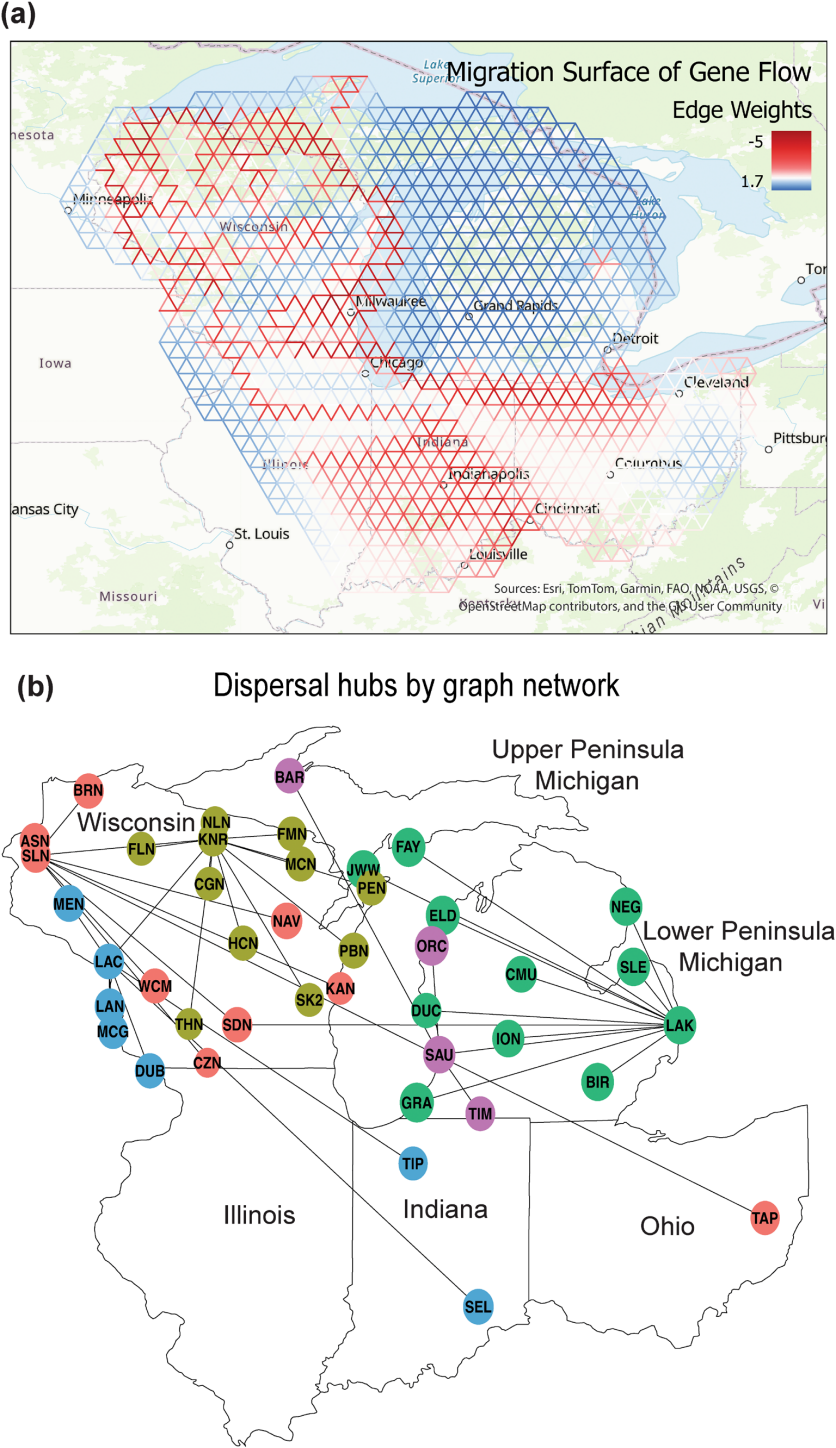
Panel (a) represents the time‐averaged, effective migration surface with edge weights, which describes gene flow patterns across Wisconsin, Illinois, Indiana, Michigan, and Ohio in the Midwest. Hues of red color indicate areas of below‐average gene flow (i.e., genetic barriers), and blue hues indicate areas of above‐average gene flow (i.e., genetic connectivity). Wisconsin exhibits spatial heterogeneity in genetic barriers, whereas Michigan displays the greatest homogeneity in genetic connectivity. Panel (b), a graph network constructed using Dps as the genetic distance, indicates hubs of dispersal across the Midwestern region. Hubs in northern Wisconsin and Eastern Michigan are clear.

To examine the time‐averaged and heterogeneous IBD pattern of gene flow across our sample sites, we generated an effective migration surface (Figure 4a). Wisconsin contains spatial heterogeneity in genetic barriers (red‐hued regions), while Michigan is relatively homogeneous in genetic connectivity (blue‐hued regions). For Wisconsin, in particular, barriers surround most northern Wisconsin populations. In southern and central Wisconsin, there are a few east–west gene flow corridors, possibly connecting to Illinois. A long corridor connecting Illinois, Indiana, and Ohio can be found, but the white color indicates a lack of gene flow certainty. We found evidence for a contiguous barrier that isolates Michigan from the rest of the Midwest. Migration surfaces with other lambda values all agree on the separation of Michigan from other regions (Figure S6). Besides range‐wide connectivity and barriers, examining whether specific sites and areas act as a hub of genetic connectivity to other sites is also helpful. The Dps‐based genetic graph, which reveals more recent gene flow, indicates two hubs in northwestern and northern Wisconsin and another hub in eastern Michigan (Figure 4b). Indiana and Ohio are more connected to Wisconsin than to Michigan. Lastly, the Wisconsin–Iowa border's Mississippi River region and Indiana populations constitute their own dispersal network.

**FIGURE 5:**
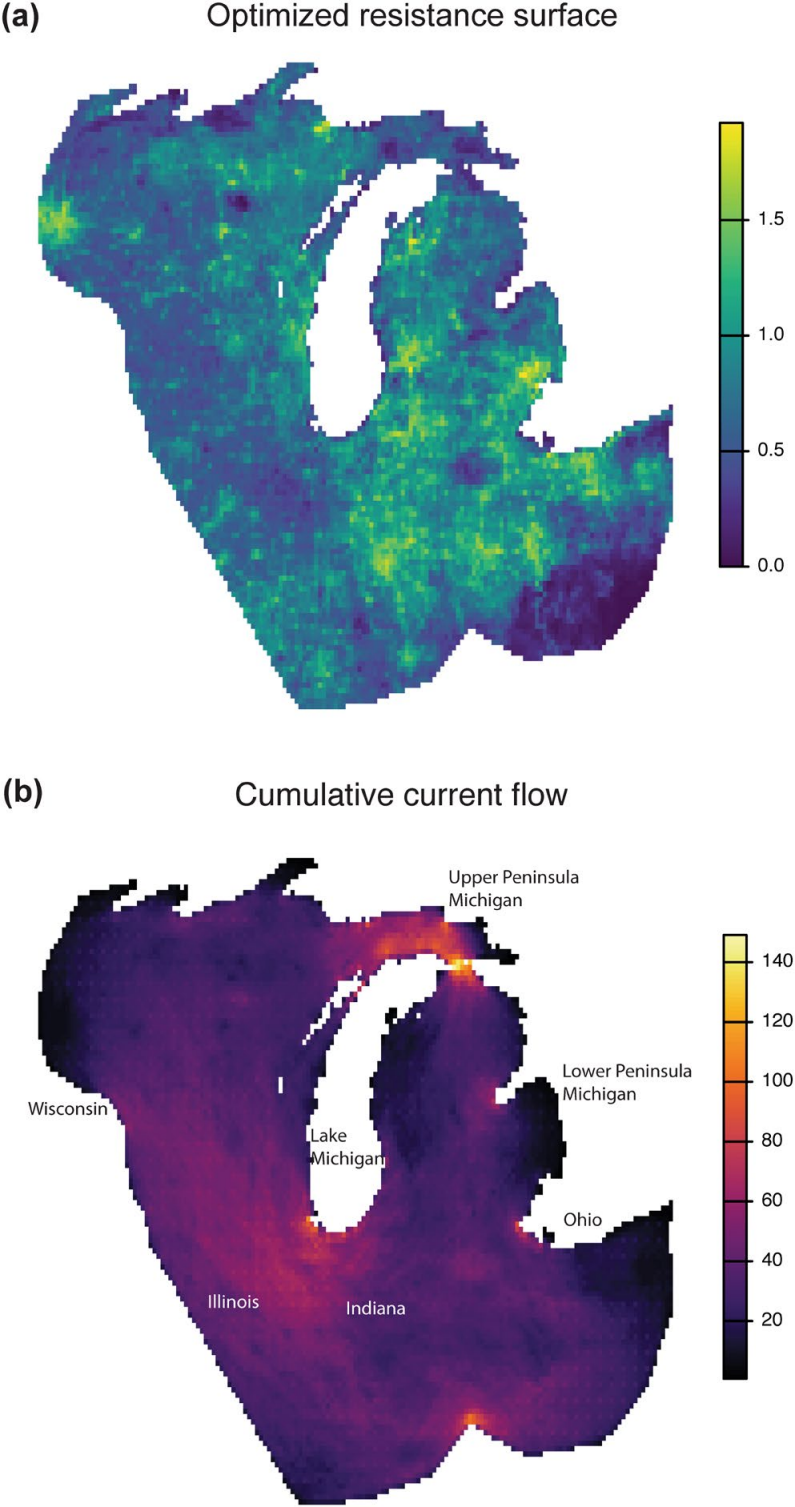
Inferred resistance landscape and current flow in the Midwest based on landscape variables. Panel (a) shows the logged optimized resistance surfaces calculated using three genetic distance metrics. Brighter color indicates areas with greater resistance to gene flow. Panel (b) illustrates the inferred current flow over the resistance landscape as a proxy for gene flow. Brighter color indicates areas with more gene flow.

The previous gene flow model is agnostic to the mechanism behind gene flow. In order to assess the effects of the multivariate landscape modulating gene flow between populations at sampling locations, we measured genetic Isolation by landscape resistance (IBR) across the genetic distance metric linearized Fst using *RADISH*. The best IBR model among all possible landscape combinations (Tables 1, S6) suggests that soil organic matter and clay content, precipitation of the driest quarter, and solar radiation have significantly positive influences on the modeled conductance (or gene flow), while human footprint has a significant negative effect. Among the significant predictors, the level of soil organic matter has the greatest influence on conductance. The IBR model performs better than the IBD null model, according to AIC, and explains 27% of the genetic variance. Furthermore, the log‐optimized resistance surface, derived from the IBR result, shows that the Indiana and Ohio regions contain areas of high resistance (Figure 5a). The cumulative current flow based on the optimized resistance surface (Figure 5b) shows significant flow at the northern Illinois and Indiana region and the Straits of Mackinac region connecting Michigan's Upper and Lower Peninsulas.

Next, we tested the IBE landscape model as an alternative model to examine the contributions of at‐site multivariate landscape conditions to the measured genetic distance among populations using *MMRR* modeling. During IBE testing, we accounted for the contribution of either geographic or resistance distance (either IBE + IBD or IBE + IBR) (Tables S7, S8). Results show that the IBE + IBD model explains 21% of the genetic variance while the IBE + IBR model explains 33% (Table 1). IBE models explain some additional variance, but the contributions are quite small compared to the contribution of IBR on top of IBD.

Lastly, in order to account for any effect of deeper ancestral population structure and objectively select the best model for biological interpretation, we used LMM and extracted AIC_*C*_ to compare across IBD, IBR, and IBE models. Results show that IBR outperforms IBD and IBE models, and IBE models are no longer significant (Tables 1, S9). The IBR model will be used to interpret the biological system.

## 4 Discussion

We set out to reconstruct the range expansion events of blacklegged ticks in Wisconsin, Michigan, and nearby regions, using population genomic variation and dense spatial sampling. Our data analyses revealed genetic and landscape connectivity patterns not known in the existing literature. We tested the hypotheses that (1) the expansion of blacklegged ticks in Wisconsin is from local northwestern refugia, while (2) the recent expansion in Michigan is derived originally from populations in Wisconsin. Additionally, we tested the hypothesis that (3) connectivity is driven primarily by geographical distance and is not principally explained by landscape variation.

Wisconsin blacklegged ticks have genomic diversity patterns suggesting that statewide range expansion began in two regions: one in northern Wisconsin and the other from the Mississippi River riparian area in southwestern Wisconsin. The first region is consistent with the northwestern local refugia re‐emergence hypothesis. The genomic variation in southwestern Wisconsin populations resembles other blacklegged ticks from southern states in the Midwest (e.g., Indiana), suggesting populations have been present in the riparian region and interconnected across the southern Midwestern region for an extended period. For the second hypothesis, our results show that, contrary to the existing perception, the Wisconsin range expansion likely did not enter extensively into Michigan. This finding is new to the literature, so our study design does not clearly determine where the Michigan range expansion originated. Some of our results suggest areas around or beyond Eastern Michigan were initially colonized. For the third hypothesis, results show that while geographical IBD drives connectivity, landscape heterogeneity further constrains the genetic connectivity of blacklegged ticks, despite the limited impact on overall genetic variation. Landscapes with high soil humidity during summertime and limited human footprints generally provide the greatest gene flow among populations.

### 4.1 Gene Flow Patterns of Blacklegged Ticks in the Midwest

Genetic differentiation within the Midwestern region is limited due to the recent population expansion events throughout the region (Table S4), but populations within the Midwest are more heterogeneous than previous genomic research suggested (Frederick et al. 2023; Schoville et al. 2024). Genetic differentiation of individual samples, categorized by sampling region, is slightly partitioned beyond panmixia in the PCA (Figure A2). The genetic differentiation and spatial patterns are more clearly visualized by population structure in ancestry coefficients (Figure 3). In particular, Wisconsin has a unique ancestry that is not found in other Midwestern populations (*K* = 3), suggesting that refugia re‐emergence contributed to unique genetic diversity in blacklegged tick range expansion events limited within Wisconsin (Figure 3). The population from Ohio has indistinct genomic ancestry but has the closest resemblance to Wisconsin populations (Figures 3, S5c). The resemblance may be because Ohio contains rare ancestral variation not found in other populations in the region, possibly from western Appalachia (Frederick et al. 2023; Schoville et al. 2024). Ohio, with unique variants, indicates that future research can identify the eastern limit of Midwestern gene flow.

While Midwestern populations in Wisconsin and Michigan are undergoing range expansion (Figure 2c), they contain noticeable spatial genetic differences, with less diversity in Michigan (Figure 2b). This is consistent with survey data showing that Michigan has been recently invaded (since at least 1998) by blacklegged ticks, four decades later than the earliest Wisconsin records (Lantos et al. 2017; Walker et al. 1998). However, the existence of private alleles in Michigan suggests that Michigan's recent blacklegged tick emergence and establishment is likely not rooted in dispersal from Wisconsin, Indiana, or Ohio. In fact, several analyses reveal a break in gene flow patterns between Michigan and other Midwestern regions. Michigan is genetically homogeneous and distinct in the clustering analysis (Figures 3, S5), even when different methods or varying assumptions about the number of ancestral clusters (*K*) are tested. Michigan also appears isolated in the migration surface and graph network analyses (Figure 4). In the graph network, Michigan comprises its own connectivity modules, which are mostly not shared with other regions. Therefore, Wisconsin, Indiana, and Ohio are less likely sources for the invasion in Michigan based on our current sampling and analytical results. Future sampling in eastern Indiana and western Ohio may help elucidate this interpretation.

Taken together, these analyses suggest that most Michigan blacklegged ticks are not derived from the Midwest region. We conducted sample collection in the Midwest (eastern North Central) region with reasonable effort, following prevailing hypotheses based on surveillance data. This finding is new to the literature and unexpected because previous work suggested that Michigan had been invaded by neighboring states from the west (Eisen and Eisen 2023), and the main question has been whether Michigan ticks have invaded more from Indiana or northern Wisconsin (Lantos et al. 2017). At the same time as the Michigan invasion (since the mid‐1990s and early 2000s), blacklegged ticks were expanding in southeastern Canada (Crandall et al. 2024; Eisen and Eisen 2023; Lindsay et al. 1999; Ogden et al. 2006). The blacklegged tick invasion in Canada led to an increasing abundance in southern Ontario, which is geographically close to Michigan (Nelder et al. 2021). Interestingly, the graph network result indicates that Lakeport State Park Campground (LAK) in lower eastern Michigan, bordering Ontario, is a major dispersal hub for the recent range expansion within Michigan. No existing literature has studied the possibility of invasion from Ontario to Michigan, as it is misaligned with the existing understanding of the invasion timelines of the two regions. Nevertheless, we suggest future research address this new hypothesis more directly by sampling populations in Ontario to test possible source areas (Krakowetz et al. 2011).

### 4.2 Significant Landscape Influence on Region‐Wide Gene Flow

Landscape heterogeneity is an essential modifier in the dynamics of range expansion by modulating favorable or unfavorable resource and spatial configurations that influence dispersal (Miller et al. 2020). Our study shows that landscape heterogeneity (IBR), in addition to dispersal constraint over large distances (IBD), influences blacklegged tick isolation (Tables 1, S6). Blacklegged tick dispersal success is reduced while traversing habitats that differ drastically in humidity during July and August, when precipitation is the lowest in the Midwest (Zhou et al. 2022). This result is consistent with field studies that show questing (host‐seeking) and dispersing ticks experience high mortality under dry conditions (Berger et al. 2014; Thomas et al. 2020). In other words, less disturbed habitat corridors with high soil organic matter and clay content, which produce humid soil conditions after rainfall, are conducive for dispersal.

The landscape‐informed gene flow patterns were investigated using the resistance model (IBR), which explains the most genetic variation, i.e., with linearized Fst (Table 1). Our analysis suggests that the favorable landscape around the Straits of Mackinac connecting Michigan's Upper Peninsula to the Lower Peninsula contributes to blacklegged ticks' genetic connectivity across Michigan (Figures 4, 5b). Since the Strait is disconnected by a water body, it may be reasonable to suspect avian hosts as the main driver of gene flow. In addition, the genetic graph network suggests that tick populations from Michigan might disperse readily into the Upper Peninsula as the most parsimonious scenario rather than the reverse (Figure 4b). To the south, northern Indiana and Illinois seem to have high‐quality environments that facilitate gene flow among all Midwestern blacklegged ticks (Figure 5b).

However, the landscape corridor that connects Michigan with Indiana does not align with our interpretation of Michigan's genetic isolation. One way to reconcile this incongruence is to examine the ancestral admixture plot, which reveals Sandburg (SDN), one population in southern Wisconsin, sharing a similar ancestry pattern with populations in Michigan (Figure 3). This standalone site might statistically support the current flow that indicates a corridor through southwestern Michigan (Figure 5b). In fact, the SDN population, along with a number of individuals from other Wisconsin populations (Figure A3), carries the Michigan ancestry pattern, suggesting the possible entry of Michigan ticks into Wisconsin, in addition to Michigan's separate genetic source. Nevertheless, these minor signals cannot compete with the dominant interpretation of population‐based and regional gene flow patterns.

Another challenge of our landscape‐informed gene flow model is that the landscape resistance takes a region‐wide average and does not capture the local dynamics, such as the varying environment–gene‐flow relationships that blacklegged ticks exhibit as host generalists with their divergent host networks (Guerra et al. 2002; Keirans et al. 1996; Lee et al. 2014; Rydzewski et al. 2011). For example, in Michigan, the landscape resistance model shows areas of high resistance (Figure 5a), while the effective migration surface, a landscape‐agnostic genetic coalescence model, shows high gene flow throughout Michigan (Figure 4a). The region‐wide averaged landscape resistance model may have taken the high landscape resistance values driven by the ticks' unique host networks in Wisconsin and overrepresented resistance in Michigan. Alternatively, Michigan populations are likely not yet in migration‐drift equilibrium, and gene flow from other regions may still be occurring (Landguth et al. 2010). Perhaps most importantly, it should be recognized that the landscape resistance model only explains 27% of the total genetic variation, which means other factors besides landscape variation, such as genetic drift, play an important role.

Lastly, although the IBE models explain additional genetic variance (Table 1), they became statistically insignificant after correcting for ancestral population structure (Table S9), which indicates the lack of interpretability of their results. It is important to note, however, that the ancestry‐pair adjustment is categorical in nature. When the categorical readjustment attempts to control for admixed historical population structures, it may have diminished meaningful among‐region environmental differences that the IBE models are designed to detect. Nevertheless, it is clear that the blacklegged tick's dispersal is less constrained by local habitat conditions and more constrained by the landscape resistance impacting host dispersers.

### 4.3 Dynamics of Range Expansion

Range expansion can be dynamic, and multiple processes may happen simultaneously. The range expansion process in natural populations is often categorized based on their dynamics as being pulled, semi‐pushed, or pushed (Birzu et al. 2018, 2021). These dispersal modes are determined by whether low‐density dispersers at the front of the expansion stochastically occupy new habitats (more pulled‐like) or if high‐density dispersers move continuously into the expansion front (more pushed‐like) (Birzu et al. 2018, 2021). Typically, pulled populations undergo more substantial genetic drift that results in more remarkable allele frequency changes (allele surfing), while pushed populations are characterized by similarly high levels of genetic diversity throughout the expanded range (Excoffier et al. 2009; Williams et al. 2019).

Our genetic data suggest that invasive populations in Michigan exhibit “pulled” dynamics, with most individuals containing fewer rare variants. However, there is somewhat limited genetic diversity to begin with, similar to the expansion dynamic at the northern edge of blacklegged tick spread in Canada (Talbot et al. 2020). Meanwhile, Wisconsin populations exhibit more “pushed” dynamics, where recent re‐emergence from possible refugia has contributed to populations across the state so that they have retained more genetic diversity and rare allelic variation (Schoville et al. 2024; Williams et al. 2019). More thorough evolutionary modeling is needed to estimate the dispersal rate in each region.

One key factor that drives range expansion dynamics in ticks is the movement of host species. For questing blacklegged ticks that utilize a variety of hosts, the patterns of gene flow across states and within states revealed in our study could be partially due to the difference in abundance and movement of hosts transporting ticks. White‐footed mouse ( *Peromyscus leucopus* ) and white‐tailed deer ( *Odocoileus virginianus* ) are the most common hosts in woodland habitats (Larson et al. 2021, 2022), whereas migratory birds are speculated to facilitate long‐distance dispersal (Ogden et al. 2015; Sidge et al. 2021; Talbot et al. 2020; Tsao et al. 2021). Our results inherently reflect the relative importance of different host dynamics since genomic diversity at sites is the product of time‐averaged gene flow across the landscape. While it remains unclear how the dynamics of different hosts might explain our observed data, we note a large 300 km genetic dispersal limit (Figure S3, see also (Schoville et al. 2024)) and sharp genetic differentiation at some geographically close populations. These patterns suggest that direct long‐range dispersers are important throughout the Midwest. A current roadblock to disentangling the contribution of hosts is the lack of detailed spatial maps of host abundance and connectivity for the entire Midwest that could be used to test alternative demographic models. Developing such resources would advance future range expansion modeling.

### 4.4 Vector Management Implications

Although over the Midwestern region under study, blacklegged ticks have spread quickly since the 1970s and are now widespread in the region, a few counties and localities are still recalcitrant to blacklegged tick establishment and naïve to the pathogens it brings (Centers for Disease Control and Prevention 2024, 2025). Identifying management units and parsing out gene flow mechanisms remain helpful strategies to slow the current spread to unestablished regions or relieve the already established regions (Hemming‐Schroeder et al. 2020; Schmidt et al. 2021).

Regarding genetic management units, it is noteworthy that the rapid invasion does not erase the signal of ancestries that are distinctive in each Midwestern state (Figure 3). The separate ancestries suggest that the current range expansion may come from multiple expansion fronts, including northern Wisconsin refugia and two other identified dispersal hubs. In designating management units, it is crucial to consider these tick populations separately, mediated by potentially different host networks. Identifying and constraining those host networks may help reinforce the separation of these clusters and promote inbreeding. Genetic homogenization may be beneficial for vector control, making them more susceptible to targeted pesticide control and stochastic population decline (Athrey et al. 2012).

Regarding mechanisms that drive gene flow, the landscape factors provide connectivity within states and at some state boundaries. For the landscape‐specific corridor at the Straits of Mackinac area in Michigan (Figure 5b), we know that this area contains high soil humidity in dry seasons with limited human impacts, and these conditions can drive dispersal (Table 1). It may be essential to examine how the favorable habitats around the Straits of Mackinac can overcome the disconnection created by the water body, such as through dispersal mechanisms facilitated by avian hosts. Similarly, the northern Illinois–Indiana border region, although predominantly agricultural and industrial, may contain highly suitable corridors for tick populations to disperse. Within Wisconsin, the region with the most gene flow heterogeneity, it is important to investigate the east–west gene flow corridors revealed by the migration surface (Figure 4a). Researchers and public health workers may examine what non‐landscape factors contribute to the connectivity, such as non‐terrestrial host networks. Further research could examine whether targeted deer control or aerial treatment of a forest swath at those corridors may slow dispersal. As Lyme disease and other vector‐associated diseases usually follow the establishment of blacklegged ticks, we may see some public health benefits from such a spatial genetics approach to tick management.

Lastly, landscape factors, such as cover type and configuration, climatic conditions, and habitat quality, only significantly explain a minor proportion of genetic variation and gene flow (Table 1). Researchers and public health workers should be informed that (1) ticks may also persist and disperse across less favorable landscapes, so it is best to assume tick presence and disease risk in some seemingly unfavorable habitats. (2) Although challenging and contentious, landscape‐agnostic methods to control the spread of blacklegged ticks, such as constraining or intervening in host networks, could theoretically minimize the stochastic spread of blacklegged ticks in the study region.

## 5 Conclusions

This region‐scaled population genetic study reveals that Midwestern blacklegged tick populations, despite recent invasion, exhibit more genetic structure than previously appreciated. Our results support previous speculation that blacklegged ticks expanded their range within Wisconsin from northern refugia. At the same time, another southwest Wisconsin ancestral cluster has been identified in the Mississippi watershed, which deserves future exploration. Contrary to our previous understanding, Michigan receives limited gene flow from its geographical neighbors in the Midwestern United States, which prompts the testing of alternative hypotheses regarding contemporary and historical gene flow from populations outside the region. The coupling of fine‐scale population genetics with landscape ecological data also revealed that landscapes with high soil humidity during the summertime, accompanied by limited human activity, could promote gene flow among blacklegged ticks in natural areas. We identified some functional corridors for future studies and management interventions. However, it is essential to note that the strength of the landscape genetic relationship is limited, given the geographical scope of the research and the rapid range expansion of blacklegged ticks.

This research is the first to visualize the heterogeneous pattern of gene flow at a regional scale and reveal significant among‐site landscape impacts on the gene flow of blacklegged ticks. The diversity of spatial patterns of gene flow over the past few decades may serve as a valuable model system, prompting further basic research into the nuanced process of range expansion. From a public health perspective, the relative genomic isolation at larger spatial scales allows for independent regional management of the spread of Lyme disease, but the high connectivity seen in habitats at local scales makes on‐the‐ground management of public and private lands more challenging.

## Author Contributions

**Dahn‐young Dong:** conceptualization (equal), data curation (lead), formal analysis (lead), investigation (lead), methodology (lead), project administration (lead), supervision (supporting), visualization (lead), writing – original draft (lead), writing – review and editing (lead). **Susan M. Paskewitz:** funding acquisition (equal), investigation (supporting), project administration (supporting), resources (equal), writing – original draft (supporting), writing – review and editing (supporting). **Jean I. Tsao:** investigation (supporting), project administration (supporting), resources (equal), writing – original draft (supporting), writing – review and editing (equal). **Sean D. Schoville:** conceptualization (equal), funding acquisition (lead), investigation (supporting), methodology (supporting), project administration (supporting), resources (equal), supervision (lead), writing – original draft (supporting), writing – review and editing (equal).

## Conflicts of Interest

The authors declare no conflicts of interest.

## Supporting information


**Data S1:** ece372360‐sup‐0001‐DataS1.docx.

## Data Availability

All raw sequence data are publicly available in the SRA database at NCBI (BioProject PRJNA1193100). Scripts, genotype data, and input data that were used to generate analysis results are available on Dryad (DOI: 10.5061/dryad.c866t1gh7).
