# Supplementary material for: Genetic and Landscape Connectivity of Blacklegged Ticks During Range Expansion in Select States of the Midwestern USA
Source: Ecol Evol. 2025 Oct 22;15(10):e72360. doi: 10.1002/ece3.72360 (PMC12541286; doi:10.1002/ece3.72360)
Supplement: Supplementary file 1 — Data S1: ece372360‐sup‐0001‐DataS1.docx. [file ECE3-15-e72360-s001.docx]

**Supplemental Information for:**

**Genetic and landscape connectivity of blacklegged ticks during range expansion in select states of the Midwestern U.S.**

Dahn-young Dong^1^, Susan M. Paskewitz^2^, Jean I. Tsao^3,4^, Sean D. Schoville^2*^

[Table S 1. Full names of the sampling sites, corresponding acronyms shown in Figure 1, and associated geographic/administrative regions. 3](#_Toc210640885)

[Table S 2. A list of Mantel tests with Pearson correlation using various genetic distances and Great Circle geographic distance, to test Isolation by Distance (IBD). 5](#_Toc210640886)

[Table S 3. The summary of genomic reads. 5](#_Toc210640887)

[Table S 4. Summary statistics of the population-based pairwise genetic distances calculated. 5](#_Toc210640888)

[Table S 5. The correlation matrix of various genetic distances 6](#_Toc210640889)

[Table S 6. Isolation by Resistance model selection based on AIC values. For each genetic distance, the three best and worst fitting models are shown. In addition, IBD null models are included for comparison. The top model in each genetic distance metric is bolded, where the landscape features are appended with their modelled coefficient estimates and significant values. Positive estimates in the RADISH model suggest increasing conductance, or promoting gene flow. Significance codes: 0 ‘***’ 0.001 ‘**’ 0.01 ‘*’. 7](#_Toc210640890)

[Table S 7. *MMRR* testing of Isolation by Environment while accounting for Isolation by Distance using three genetic distance metrics. 8](#_Toc210640891)

[Table S 8. *MMRR* testing of Isolation by Environment while accounting for Isolation by Resistance using three genetic distance metrics. 10](#_Toc210640892)

[Table S 9. MLPE tests to control for historical population structure (PS), *K* = 3, as a random effect for the landscape genetics models 12](#_Toc210640893)

[Figure S 1. For demonstration purposes and method clarification purposes only. Population structure analyses and genomic summary statistics after applying Minor Allele Count = 2 filter in addition to the steps described in the Method, effectively removing singleton alleles. The results quantitatively resemble the original analyses without applying minor allele filters. And the interpretations of these results remain the same. Panel 1 is a PCA plot of individual genomic samples categorized by regions. Panel 2 is SNMF plots of ancestry coefficients of grouped populations at the location of sampling. Panel 3 is population-based genomic diversity and summary statistics. These plots demonstrate that it is reasonable to skip MAC filtering in the data preparation stage to retain high-coverage singletons. 15](#_Toc210640894)

[Figure S 2. Isolation by distance plots: genetic distances measured as (a) linearized Fst, (b) Dps, or (c) PG against geographic distance in meters. 16](#_Toc210640895)

[Figure S 3. Mantel correlogram of the genetic distance measured as Dps, PG, or linearized Fst against Great Circle distance. The pattern of Isolation by distance becomes stronger beginning about 300 kilometers. The distance where IBD begins is 324 km for Dps, 341 km for PG, and 335 km for linearized Fst. 18](#_Toc210640896)

[Figure S 4. Cross-entropy comparison of model fit for varying levels of population structure (K values) for *SNMF* analysis shown in Figure 3 and Figure S5. The minimal cross-entropy value suggests a better fit, and *K* = 2 is the best and *K* = 3 is the second. 19](#_Toc210640897)

[Figure S 5. *CONSTRUT* results (a) comparing spatial models that account for isolation by distance and non-spatial models. (b) Layer contribution to ancestry proportion. Any *K* larger than 3 contains a layer that yields less than 1% to an ancestry, limiting biological interpretation. (c) Spatial model with *K* = 3, resembling the result of the *K* = 3 *SNMF* model. 20](#_Toc210640898)

[Figure S 6. *FEEMS* migration surface results using different cross-validated lambda values. 21](#_Toc210640899)

Table S 1. Full names of the sampling sites, corresponding acronyms shown in Figure 1, and associated geographic/administrative regions.

| Site Full Name | Acronym | Region |
| --- | --- | --- |
| Selmier State Forest | SEL | Indiana |
| Tippecanoe River State Forest | TIP | Indiana |
| Baraga State Park | BAR | Upper Peninsula Michigan |
| Bird Hills Nature Area | BIR | Lower Peninsula Michigan |
| CMU Neithercut Woodland | CMU | Lower Peninsula Michigan |
| Duck Lake State Park | DUC | Lower Peninsula Michigan |
| El Dorado Park | ELD | Lower Peninsula Michigan |
| Fayette Historic State Park | FAY | Upper Peninsula Michigan |
| Grand Mere State Park | GRA | Lower Peninsula Michigan |
| Ionia State Recreation Area | ION | Lower Peninsula Michigan |
| JW Wells State Park | JWW | Upper Peninsula Michigan |
| Lakeport State Park Campground | LAK | Lower Peninsula Michigan |
| Negwegon State Park | NEG | Lower Peninsula Michigan |
| Orchard Beach State Park | ORC | Lower Peninsula Michigan |
| Saugatuck Dunes State Park | SAU | Lower Peninsula Michigan |
| Sleeper State Park | SLE | Lower Peninsula Michigan |
| Timm Preserve County Park | TIM | Lower Peninsula Michigan |
| Tappan Lake Park | TAP | Ohio |
| Amsterdam Slough | ASN | Wisconsin |
| Brule | BRN | Wisconsin |
| Cadiz Springs | CZN | Wisconsin |
| Council Grounds | CGN | Wisconsin |
| Flambeau ATV Trails | FLN | Wisconsin |
| Fox Maple | FMN | Wisconsin |
| Hartman Creek | HCN | Wisconsin |
| Kemp Natural Resource Research Station | KNR | Wisconsin |
| Kohler Andrae | KAN | Wisconsin |
| La Crosse | LAC | Wisconsin |
| Lake Menomin Park | MEN | Wisconsin |
| McClintock | MCN | Wisconsin |
| Navarino | NAV | Wisconsin |
| North Trout Lake | NLN | Wisconsin |
| Peninsula State Park | PEN | Wisconsin |
| Point Beach | PBN | Wisconsin |
| Sandburg | SDN | Wisconsin |
| South Kettle Moraine State Park | SK2 | Wisconsin |
| Straight Lake | SLN | Wisconsin |
| Tower Hill | THN | Wisconsin |
| Wildcat Mountain State Park | WCM | Wisconsin |
| Dubuque | DUB | Wisconsin-Iowa border |
| Lansing | LAN | Wisconsin-Iowa border |
| McGregor | MCG | Wisconsin-Iowa border |

Table S 2. A list of Mantel tests with Pearson correlation using various genetic distances and Great Circle geographic distance, to test Isolation by Distance (IBD).

|  | Mantel correlation | p_value |
| --- | --- | --- |
| Linearized fst | 0.27585641 | 0.001 |
| PG | 0.37636975 | 0.001 |
| Dps | 0.21042542 | 0.001 |
| Jost | 0.23616001 | 0.003 |
| Hedrick | 0.20503036 | 0.015 |
| NeiGst | 0.20215223 | 0.017 |
| Nei | 0.1501539 | 0.049 |
| Reynolds | 0.12512457 | 0.113 |
| rare_allele_weighted | 0.01351253 | 0.408 |

Table S 3. The summary of genomic reads.

|  | Single Nucleotide Polymorphisms (SNPs) after filtering for variant missingness | SNPs after accounting for Linkage disequilibrium |
| --- | --- | --- |
| All samples across regions (n=517) | 112791 | 79815 |

Table S 4. Summary statistics of the population-based pairwise genetic distances calculated.

|  | Min. | 1st Qu. | Median | Mean | 3rd Qu. | Max. |
| --- | --- | --- | --- | --- | --- | --- |
| Linearized fst | 0 | 0.01 | 0.02 | 0.027 | 0.043 | 0.083 |
| Dps | 0.015 | 0.02 | 0.022 | 0.022 | 0.024 | 0.03 |
| PG | 0.251 | 0.747 | 0.916 | 0.916 | 1.079 | 1.726 |
| Nei | 0.001 | 0.002 | 0.003 | 0.003 | 0.004 | 0.007 |
| Reynolds | 0.178 | 0.236 | 0.278 | 0.282 | 0.32 | 0.437 |
| rare_allele_weighted | 36.144 | 44.529 | 47.214 | 48.644 | 51.998 | 72.871 |
| Jost | 0 | 0.001 | 0.001 | 0.002 | 0.002 | 0.004 |
| Hedrick | 0.001 | 0.016 | 0.037 | 0.039 | 0.058 | 0.103 |
| NeiGst | 0 | 0.008 | 0.018 | 0.019 | 0.029 | 0.052 |

Table S 5. The correlation matrix of various genetic distances

|  | Nei | Reynolds | Dps | PG | rare_allele_weighted | Linearized fst | Jost | Hedrick | NeiGst |
| --- | --- | --- | --- | --- | --- | --- | --- | --- | --- |
| Nei | / | / | / | / | / | / | / | / | / |
| Reynolds | 0.91 | / | / | / | / | / | / | / | / |
| Dps | 0.89 | 0.64 | / | / | / | / | / | / | / |
| PG | 0.45 | 0.24 | 0.61 | / | / | / | / | / | / |
| rare_allele_weighted | 0.76 | 0.67 | 0.65 | 0.21 | / | / | / | / | / |
| Linearized fst | 0.77 | 0.64 | 0.8 | 0.58 | 0.23 | / | / | / | / |
| Jost | 0.9 | 0.87 | 0.77 | 0.48 | 0.42 | 0.92 | / | / | / |
| Hedrick | 0.87 | 0.93 | 0.66 | 0.36 | 0.43 | 0.83 | 0.98 | / | / |
| NeiGst | 0.87 | 0.93 | 0.65 | 0.35 | 0.43 | 0.83 | 0.97 | 1 | / |

Table S 6. Isolation by Resistance model selection based on AIC values. For each genetic distance, the three best and worst fitting models are shown. In addition, IBD null models are included for comparison. The top model in each genetic distance metric is bolded, where the landscape features are appended with their modelled coefficient estimates and significant values. Positive estimates in the RADISH model suggest increasing conductance, or promoting gene flow. Significance codes: 0 ‘***’ 0.001 ‘**’ 0.01 ‘*’.

| Genetic distance models and ranking | Landscape features and significant estimates of the best model | Optimized IBR models | |
| --- | --- | --- | --- |
|  |  | AIC | Delta AIC |
| **Linearized Fst-1** | Clay (0.4 ***) + Soil organic matter (0.8 ***) + Human footprint (-0.4 **) + Solar radiation (0.4 *) + Precipitation of driest quarter (0.4 *) | -6274.649 | 0 |
| Linearized Fst-2 | Clay + Soil organic matter + Human footprint + Tree cover + Solar radiation + Precipitation of driest quarter | -6272.694 | 1.95 |
| Linearized Fst-3 | Clay + Soil organic matter + elevation + Human footprint + Solar radiation + Precipitation of driest quarter | -6272.667 | 1.98 |
| Linearized Fst-IBD | Geographic distance | -6243.276 | 31.4 |
| Linearized Fst-255 | Clay + Tree cover + Solar radiation + Precipitation of driest quarter | -6237.660 | 37.0 |
| **PG-1** | Soil organic matter (0.9 ***) + Human footprint (-0.4 *) + Tree cover (-0.8 ***) + Precipitation of driest quarter (0.4 *) | -2053.692 | 0 |
| PG-2 | Soil organic matter + Human footprint + Tree cover + Solar radiation + Annual precipitation | -2052.817 | 0.87 |
| PG-3 | Clay + Soil organic matter + Human footprint + Tree cover + Precipitation of driest quarter | -2052.760 | 0.93 |
| PG-IBD | Geographic distance | -2035.099 | 18.6 |
| PG-255 | Tree cover + Precipitation of driest quarter | -2032.684 | 21.0 |
| **Dps-1** | Soil organic matter (1.2 ***) + Human footprint (-0.4 **) + Tree cover (1.0 ***) + Solar radiation (2.2 ***) + Annual precipitation (-1.0 ***) + Precipitation of driest quarter (2.3 ***) | -9728.272 | 0 |
| Dps-2 | Soil organic matter + Elevation + Human footprint + Tree cover + Solar radiation + Annual precipitation + precipitation of driest quarter | -9727.403 | 0.87 |
| Dps-3 | Clay + Soil organic matter + Human footprint + Tree cover + Solar radiation + Annual precipitation + Precipitation of driest quarter | -9727.330 | 0.94 |
| Dps-IBD | Geographic distance | -9690.336 | 37.9 |
| Dps-255 | Clay + Tree cover + Solar radiation + Precipitation of driest quarter | -9685.465 | 42.8 |

Table S 7. *MMRR* testing of Isolation by Environment while accounting for Isolation by Distance using three genetic distance metrics.

| Vars tested with Linearized Fst | estimate | p |
| --- | --- | --- |
| **annual_ppt** | **0.12** | **0.04** |
| clay | -0.04 | 0.32 |
| **elevation** | **0.17** | **0.01** |
| **geodist** | **0.27** | **0** |
| human_footprint | 0.01 | 0.86 |
| Intercept | 0 | 0.07 |
| ppt_driest_quarter | 0.08 | 0.19 |
| **soil_organic_matter** | **0.17** | **0.01** |
| solar_radiation | -0.20 | 0 |
| tree_cover | 0 | 0.93 |
| **R^2^:** | **0.21** | / |
| F-Statistic: | 25.22 | / |
| F p-value: | 0 | / |

| Vars tested with Dps | estimate | p |
| --- | --- | --- |
| annual_ppt | 0 | 0.99 |
| clay | 0.05 | 0.34 |
| elevation | 0.12 | 0.12 |
| geodist | 0.14 | 0.12 |
| human_footprint | -0.01 | 0.94 |
| Intercept | 0 | 0.1 |
| ppt_driest_quarter | 0.13 | 0.12 |
| soil_organic_matter | 0.09 | 0.19 |
| solar_radiation | -0.07 | 0.37 |
| tree_cover | -0.03 | 0.5 |
| **R^2^:** | **0.1** | / |
| F-Statistic: | 10.35 | / |
| F p-value: | 0 | / |

| Vars tested with PG | estimate | p |
| --- | --- | --- |
| annual_ppt | 0.12 | 0.12 |
| clay | 0.05 | 0.37 |
| **elevation** | **0.18** | **0.03** |
| **geodist** | **0.5** | **0** |
| human_footprint | -0.02 | 0.8 |
| Intercept | 0 | 0.8 |
| ppt_driest_quarter | -0.13 | 0.12 |
| soil_organic_matter | 0.05 | 0.47 |
| **solar_radiation** | **-0.20** | **0.02** |
| tree_cover | 0.03 | 0.65 |
| **R^2^:** | **0.19** | / |
| F-Statistic: | 22.35 | / |
| F p-value: | 0 | / |

Table S 8. *MMRR* testing of Isolation by Environment while accounting for Isolation by Resistance using three genetic distance metrics.

| Vars tested with Linearized Fst | estimate | p |
| --- | --- | --- |
| annual_ppt | 0 | 0.95 |
| clay | -0.05 | 0.2 |
| **elevation** | **0.2** | **0** |
| human_footprint | 0.01 | 0.88 |
| Intercept | 0 | 0 |
| ppt_driest_quarter | 0.09 | 0.1 |
| **resistance dist** | **0.46** | **0** |
| soil_organic_matter | 0.09 | 0.07 |
| **solar_radiation** | **-0.15** | **0** |
| tree_cover | -0.02 | 0.54 |
| **R^2^:** | **0.33** | / |
| F-Statistic: | 45.81 | / |
| F p-value: | 0 | / |

| Vars tested with Dps | estimate | p |
| --- | --- | --- |
| annual_ppt | -0.06 | 0.41 |
| clay | 0.05 | 0.39 |
| elevation | 0.11 | 0.19 |
| human_footprint | -0.01 | 0.88 |
| Intercept | 0 | 0.11 |
| **ppt_driest_quarter** | **0.22** | **0** |
| resistance dist | 0.08 | 0.36 |
| soil_organic_matter | 0.07 | 0.28 |
| solar_radiation | -0.02 | 0.84 |
| tree_cover | -0.04 | 0.4 |
| **R^2^:** | **0.09** | / |
| F-Statistic: | 9.67 | / |
| F p-value: | 0.01 | / |

| Vars tested with PG | estimate | p |
| --- | --- | --- |
| annual_ppt | 0.02 | 0.81 |
| clay | 0.03 | 0.56 |
| elevation | 0.16 | 0.07 |
| human_footprint | -0.02 | 0.69 |
| Intercept | 0 | 0 |
| ppt_driest_quarter | -0.02 | 0.85 |
| **resistance dist** | **0.45** | **0** |
| soil_organic_matter | 0.05 | 0.47 |
| solar_radiation | -0.12 | 0.13 |
| tree_cover | 0.02 | 0.76 |
| **R^2^:** | **0.22** | / |
| F-Statistic: | 26.1 | / |
| F p-value: | 0 | / |

Table S 9. MLPE tests to control for historical population structure (PS), *K* = 3, as a random effect for the landscape genetics models

| Isolation models | Predictors and estimates | *p* | *AIC_c_* | Conditional *R^2^* |
| --- | --- | --- | --- | --- |
| Isolation by Distance (IBD) alone (null model) | Intercept: 0.028  IBD: 0.001  PS: 0.018 | 0.002  0.005 | -6862.484 | 0.709 |
| Isolation by Resistance (IBR) alone | Intercept: 0.028  IBR: 0.001  PS: 0.018 | 0.002  0.002 | **-6865.065** | 0.704 |
| Isolation by Environment (IBE) while co-estimating IBD | Intercept: 0.028  IBE: all insignificant  IBD: 0.001  PS: 0.018 | 0.002  /  0.052 | -6731.268 | 0.709 |
| Isolation by Environment (IBE) while co-estimating IBR | Intercept: 0.028  IBE: all insignificant  IBR: 0.001  PS:0.018 | 0.002  /  0.022 | -6733.292 | 0.706 |

Panel 1

Panel 2

Panel 3

Figure S 1. For demonstration purposes and method clarification purposes only. Population structure analyses and genomic summary statistics after applying Minor Allele Count = 2 filter in addition to the steps described in the Method, effectively removing singleton alleles. The results quantitatively resemble the original analyses without applying minor allele filters. And the interpretations of these results remain the same. Panel 1 is a PCA plot of individual genomic samples categorized by regions. Panel 2 is SNMF plots of ancestry coefficients of grouped populations at the location of sampling. Panel 3 is population-based genomic diversity and summary statistics. These plots demonstrate that it is reasonable to skip MAC filtering in the data preparation stage to retain high-coverage singletons.

(a)

(b)

(c)

Figure S 2. Isolation by distance plots: genetic distances measured as (a) linearized Fst, (b) Dps, or (c) PG against geographic distance in meters.

Figure S 3. Mantel correlogram of the genetic distance measured as Dps, PG, or linearized Fst against Great Circle distance. The pattern of Isolation by distance becomes stronger beginning about 300 kilometers. The distance where IBD begins is 324 km for Dps, 341 km for PG, and 335 km for linearized Fst.

Figure S 4. Cross-entropy comparison of model fit for varying levels of population structure (K values) for *SNMF* analysis shown in Figure 3 and Figure S5. The minimal cross-entropy value suggests a better fit, and *K* = 2 is the best and *K* = 3 is the second.

(a)

(b)(c)

Figure S 5. *CONSTRUT* results (a) comparing spatial models that account for isolation by distance and non-spatial models. (b) Layer contribution to ancestry proportion. Any *K* larger than 3 contains a layer that yields less than 1% to an ancestry, limiting biological interpretation. (c) Spatial model with *K* = 3, resembling the result of the *K* = 3 *SNMF* model.

Figure S 6. *FEEMS* migration surface results using different cross-validated lambda values.
